# Organizational and Musculoskeletal Factors of Job Satisfaction and Work Ability Among Critical Care Nurses: A Multicenter Cross‐Sectional Study

**DOI:** 10.1155/nrp/4882421

**Published:** 2026-09-29

**Authors:** Beatrice Albanesi, Daniela Chiarini, Marco Clari, Maria Vittoria Picciaiola, Alessandro Godono, Giacomo Garzaro

**Affiliations:** ^1^ Department of Public Health and Pediatrics University of Torino Turin Italy unito.it

**Keywords:** critical care nurses, job satisfaction, musculoskeletal disorder, organizational factors, work ability, workforce retention

## Abstract

**Background:**

Nursing shortage is a global challenge driven by the imbalance between the number of nurses entering and leaving the profession, with job satisfaction (JS) and work ability (WA) as key retention factors influenced by organizational and health conditions, including musculoskeletal disorders (MSDs). Although critical care nurses experience high MSDs, low JS, and reduced WA, their interrelationships remain underexplored.

**Aims:**

This multicenter cross‐sectional study investigated individual, organizational, and musculoskeletal factors associated with JS and WA among intensive care unit (ICU) and emergency department (ED) nurses.

**Methods:**

Data were collected using a researcher‐developed questionnaire assessing individual, organizational, and MSD‐related variables, together with validated instruments assessing upper‐limb disability and symptoms (DASH), Work Ability Index (WAI), and JS (COPSOQ II). Multivariable regression was performed.

**Results:**

Participants reported moderate JS (3.4 ± 0.9) and good WA (41.3 ± 5.4). Lower JS was associated with ICU employment (*β* = −0.38, *p* = 0.005) and long shifts (*β* = −0.32, *p* = 0.003). Reduced WA was associated with chronic conditions (*β* = −3.14, *p* = 0.001), work adjustments (*β* = −2.58, *p* = 0.001), ICU employment (*β* = −1.38, *p* = 0.031), unit or role changes due to MSDs (*β* = −1.60, *p* = 0.015), and hip (*β* = −1.69, *p* = 0.009) or upper‐limb MSDs (*β* = −1.41, *p* = 0.006). JS and WA were also significantly associated (*β* = 0.08 for WAI in the JS model; *β* = 2.22 for JS in the WAI model).

**Conclusions:**

A multidisciplinary, multilevel, and multicomponent approach may support JS and WA and inform strategies to reduce MSD‐related burden and promote nurse retention.

## 1. Introduction

The nursing profession is currently experiencing a global workforce crisis, with a growing gap between the number of nurses entering and leaving the profession [1]. An estimated 13 million additional nurses will be required worldwide in the coming decade [2]^,^ driven by a combination of factors, including increasing demand for care, population aging, and the rising prevalence of chronic conditions [3]. The resulting shortage has fostered challenging work environments, characterized by excessive workloads, understaffing, mandatory overtime, and long working hours, in stressful and unsafe conditions [1, 3]. Consequently, beyond age‐related retirement, more nurses are voluntarily leaving the profession due to unfavorable working conditions and stressful organizational environments [4].

Among the main factors in nurse retention or turnover, job satisfaction (JS) and work ability (WA) are key, both influenced by working conditions and with significant implications for workforce stability and sustainability [1, 4]. JS reflects an individual’s overall feelings about the job, influenced not only by the nature of the work itself but also by the expectations individuals hold for what the job should provide [5]. JS is a multidimensional construct encompassing a range of individual factors, such as relationships with colleagues and opportunities for personal growth and career advancement, as well as organizational factors, including working conditions, policies, and job security [6]. WA, as defined by Tuomi and colleagues [7], describes an individual’s current and future capacity to meet job demands while considering health status, functional abilities, and mental resources. It has emerged as a strong predictor of nurses’ intention to leave the profession, particularly in the current context of an aging nursing workforce and among younger nurses [8]. Among the factors influencing WA, MSDs represent a key condition [9]. In nurses, MSDs are strongly associated with reduced WA and increased work limitations [10], which may in turn contribute to lower JS, although the direct relationship with JS remains unclear [11, 12]. Nevertheless, evidence suggests a bidirectional, reinforcing relationship between JS and WA, in which higher JS can enhance nurses’ WA, and vice versa [10].

Such knowledge is essential for targeting preventive and occupational health interventions and for strengthening the nursing workforce, particularly in high‐demand environments like critical care. Although critical care nurses are particularly exposed to high rates of MSDs, lower JS, and reduced WA [13, 14], to our knowledge, the combined relationship among these constructs remains insufficiently explored within a single analytical model. In intensive care units (ICUs) and emergency departments (EDs), these factors are further exacerbated by long shifts, excessive workloads, high patient acuity, and elevated emotional demands [15, 16].

Recent studies conducted among ICU and neonatal ICU nurses have further shown that a more positive practice environment is associated with better professional quality of life, including lower burnout and higher satisfaction, supporting the role of organizational factors in nurses’ well‐being and retention‐related outcomes [17, 18]. Therefore, a deeper understanding of how individual, organizational, and health‐related factors, including MSDs, affect JS and WA is needed to target preventive and occupational health interventions aimed at improving nurses’ well‐being, preserving WA, and supporting retention‐related outcomes, particularly in high‐demand environments such as critical care.

### 1.1. Aim

This study aimed to identify individual, organizational, and MSD‐related factors associated with JS and WA among critical care nurses. Furthermore, it seeks to explore the potential association between JS and WA.

## 2. Methods

### 2.1. Study Design and Sample

A multicenter cross‐sectional study was conducted in 10 hospitals in Northern Italy. The target population included all ICU and ED nurses employed in the participating hospitals during the study period. A total of 348 nurses who met the eligibility criteria were identified. Inclusion criteria were at least 3 years of professional experience and a work commitment corresponding to at least 75% of full‐time equivalent hours. All eligible nurses were invited to participate through institutional mailing lists. Participation was voluntary. No additional exclusion criteria were applied.

A formal a priori power analysis was not performed due to the difficulty of estimating a reliable effect size for the planned multivariable models, given the exploratory nature of the study and the lack of directly comparable literature including the same set of predictors and outcomes. Sample size adequacy was therefore evaluated according to Green’s recommendations for multivariable regression analyses [19]. Considering all candidate predictors initially planned for inclusion in the regression models, the final sample of 331 participants substantially exceeded the recommended minimum thresholds for both testing the overall regression models (*N* ≥ 50 + 8*k*) and for testing individual predictors (*N* ≥ 104 + *k*).

### 2.2. Data Collection

Data were collected between July and October 2022, using an anonymous online questionnaire. The questionnaire consisted of researcher‐designed sections developed based on relevant literature and addressing individual and work‐related factors, as well as validated instruments. The overall questionnaire underwent face and content validation by an expert panel including occupational physicians and university researchers. The panel assessed the clarity, relevance, and comprehensiveness of the items, and the questionnaire was refined accordingly. The questionnaire was structured into the following sections:

i. Individual data were collected on age, gender, and health status, such as the presence of chronic conditions and fitness for work with adjustments, while organizational variables included current work unit, any changes of unit or role due to MSDs, days of absence from work due to MSDs, average weekly working hours, and long shifts (≥ 10 h) [20]. The researcher‐developed questionnaire also collected information on MSDs occurrence during the previous 12 months and the affected body regions (e.g., back, hips, upper limbs, and lower limbs).
ii. Participants who reported upper‐limb MSDs in the researcher‐developed questionnaire subsequently completed the validated the Italian version of the Disabilities of the Arm, Shoulder and Hand (DASH) questionnaire [21] to assess upper‐limb disability and symptoms. The DASH consists of 30 items organized into three domains: (i) DASH Function/Symptom (DASH‐FS), assessing the ability to perform daily activities; (ii) DASH Work (DASH‐W), evaluating the impact of upper limb MSDs on work‐related activities, including pain intensity, pain during activity, tingling, weakness, and stiffness; (iii) DASH Sports/Performing Arts Module (DASH‐SM), assessing the impact of MSDs on participation in sports. Each item is rated on a 5‐point Likert scale, ranging from 1 (“no symptom”) to 5 (“severe symptoms”). The overall score ranges from 0 (no disability) to 100 (severe disability) [21]. The DASH demonstrated high internal consistency (*α* ≥ 0.90) [22].
iii. Data on WA were collected using the validated Italian version of the Work Ability Index (WAI) [23], which has demonstrated good internal consistency (Cronbach’s *α* = 0.72–0.80) and satisfactory test–retest reliability (*r* = 0.70–0.83) [24]. The WAI questionnaire consists of seven subscales assessing self‐rated: (i) current WA, (ii) physical and mental job demands, (iii) health conditions, (iv) sick leave, (v) perceived work impairment, (vi) mental resources, (vii) expected future WA. The WAI score ranges from 7 to 49, with higher values indicating better WA: poor (≤ 27), moderate (28–36), good (37–43), or excellent (≥ 44).
iv. JS was assessed using the Job Satisfaction Scale from the Copenhagen Psychosocial Questionnaire II (COPSOQ II) [25], which has demonstrated good internal consistency (Cronbach’s *α* = 0.80) [26]. COPSOQ II includes four items assessing satisfaction with work tasks, organization, recognition, and overall job contentment. Given the relevance of interpersonal relationships in nursing work environments, an additional item assessing satisfaction with working relationships was included by the authors of this study. All items were rated on a 5‐point Likert scale ranging from 1 (“very dissatisfied”) to 5 (“very satisfied”). The modified five‐item scale demonstrated high internal consistency in the present sample (Cronbach’s *α* = 0.89).

### 2.3. Ethical Consideration

The study was approved by the Institutional Ethical Committee (Prot. n. 0261496) and conducted in accordance with the Declaration of Helsinki. Data collection adhered fully to the General Data Protection Regulation (GDPR, EU Regulation 2016/679). All participants provided informed consent before participating voluntarily. Anonymity was ensured by pseudonymizing all data.

### 2.4. Statistical Analysis

Descriptive statistics summarized the study population. Continuous variables were reported as mean and standard deviation (SD), while categorical variables as frequencies and percentages. JS and WAI were treated as continuous outcome variables. The associations between individual, organizational, and musculoskeletal variables and JS and WAI were first explored through univariable linear regression analyses. The analytical approach was selected based on the cross‐sectional nature of the data and the study objectives, which were aimed at identifying associations among variables.

Functional impairment related to upper‐limb disorders was evaluated using the DASH modules, included as continuous variables in the regression analyses [27]. WAI categories were also examined in relation to JS. A *p*‐value of < 0.05 was considered statistically significant. Variables showing a statistically significant (*p* < 0.05) or borderline (e.g. *p* < 0.051) association in univariable analyses, as well as those reported in the literature as generally associated with our outcomes of interest, were also considered. Multicollinearity among independent variables was assessed before model fitting. Regression coefficients (*β*) and *p*‐values were reported. All analyses were performed using STATA SE/17 (StataCorp LLC, College Station, TX, USA).

## 3. Results

Overall, 348 nurses were initially considered for the study, of whom 331 participated, yielding a response rate of 95%. The mean age was 41 ± 9.7 years, and 77% were female (Table 1). The prevalence of chronic conditions was 30.8%; however, only 3.9% reported a certified disability. In our sample, 17.8% of participants required fitness for work adjustments. Most participants (44.4%) did not engage in any physical activity, while only 16% reported performing more than 3 h per week.

**TABLE 1:** Sample characteristics.

Variables	*N* (%) 831 (100%)
*Individual variables*	
Sex^∗^	
Female	254 (77.0%)
Male	76 (23.0%)
Age, years (mean ± SD)	41.3 (9.7)
< 40	146 (44.1%)
40–55	153 (46.2%)
> 55	32 (9.7%)
Cohabitation with a non‐self‐sufficient person	
No	311 (94.0%)
Yes	20 (6.0%)
Chronic conditions	
No	229 (69.2%)
Yes	102 (30.8%)
Cardiovascular diseases	
No	310 (93.7%)
Yes	21 (6.3%)
Certified disability^∗^	
No	317 (96.1%)
Yes	13 (3.9%)
Fitness for work with adjustments	
No	272 (82.2%)
Yes	59 (17.8%)
Physical activity hours per week	
No	147 (44.4%)
< 1	32 (9.7%)
1–3	99 (29.9%)
> 3	53 (16.0%)

*Organizational variables*	
Department^∗^	
ICU	268 (81.2%)
ED	62 (18.8%)
Long shifts	
No	205 (61.9%)
Yes	126 (38.1%)
Average week working hours [mean ± SD]	38.6 (3.4)
Change of unit/role due to MSD	
No	300 (90.6%)
Yes	31 (9.4%)
Absence from work due to MSDs	
No	199 (60.1%)
Yes	132 (39.9%)
Days of absence from work due to MSDs	
0	199 (60.1%)
1–7	80 (24.2%)
8–30	33 (10.0%)
> 30	19 (5.7%)

*Musculoskeletal disorders*	
Overall MSDs	
No	47 (14.2%)
Yes	284 (85.8%)
Overall back	
No	66 (19.9%)
Yes	265 (80.1%)
Lower limbs^∗^	
No	237 (71.6%)
Yes	94 (28.4%)
Upper limbs	
No	242 (75.83%)
Yes	89 (26.88%)
DASH	
Disability/symptom module	18.9 (18.7)
Work module	18.7 (25.6)
Sports/performing arts module	9.7 (20.6)

^∗^Missing Data.

Regarding organizational variables, most participants were employed in ICUs (81.2%) and worked long shifts (38.1%), with an average weekly working time of 38.6 ± 3.4 h. Over the past 12 months, 60.1% of participants reported no days of absence due to MSDs. Short‐term absences (1–7 days) occurred in 24% of cases, while only 5.7% reported absences longer than 30 days. The prevalence of MSDs in the last 12 months was 85.8%. Regarding anatomical regions, most participants reported back disorders (80.1%), followed by lower limbs (28.4%) and hip disorders (14.5%). Among the back regions, the lumbar spine was the most affected (68.9%), followed by the neck (60.1%) and dorsal regions (26.6%). Among participants reporting upper limb disorders, the mean value for the DASH‐FS module was 18.9 (SD ± 18.7), for the DASH‐W module 18.7 (SD ± 25.6), and for the DASH‐SM module 9.7 (SD ± 20.6).

The overall WAI score was 41.3 (SD ± 5.4), suggesting good WA. Most participants (49.4%) reported a WA score > 43, whereas only 2.8% reported a score < 28. Finally, regarding JS, participants reported a mean score of 3.4 (SD ± 0.9).

Among individual variables, linear regression analysis (Supporting 1: *Univariate linear regressions between JS/WAI and included variables*) showed a significant association between age and both JS and WAI. Participants aged 40–55 showed a reduction in both JS (*p* = 0.013) and WAI (*p* < 0.001), whereas in participants older than 55, the association remained significant only for WAI (*p* < 0.001). A borderline association with male gender and WAI was observed (*p* = 0.051). Regarding health status, chronic conditions and cardiovascular diseases were associated with a significant decrease in WAI (*p* < 0.001). Participants requiring fitness for work with adjustment were associated with significant decreases in both JS (*p* = 0.013) and WAI (*p* < 0.001). Being employed in ICUs was associated with lower JS (*p* = 0.004) and WAI (*p* = 0.004). A change of unit or role due to MSDs was associated with a significant reduction in JS (*p* = 0.034) and WAI (*p* < 0.001). Absence from work due to MSDs was associated with decreased JS, particularly among participants with absences of 8–30 days (*p* < 0.001) and those with long‐term absences exceeding 30 days per year (*p* = 0.012). Long shifts and weekly working hours were not associated with either JS or WAI (*p* > 0.05). Overall, MSDs reported in the past 12 months were associated with a decreased WAI (*p* < 0.001), with all body regions showing significant associations (*p* < 0.001). Specifically, back disorders were associated with both reduced JS and WAI scores (*p* < 0.001). Lower DASH‐FS and DASH‐W scores were associated with reduced JS and WAI scores (*p* < 0.001). Conversely, the DASH‐SM score was not significantly associated with either outcome (*p* > 0.05). Higher WAI scores were significantly associated with increased JS. Compared with participants in the reference group, those with WAI scores of 28–36 (*p* = 0.019), 37–43 (*p* < 0.001), and above 43 (*p* < 0.001) reported progressively higher levels of JS.

The multiple regression model (Table 2) for JS included the following variables: age, fitness for work with adjustment, department, long shift, change of unit or role, days of absence from work due to MSDs, the affected body regions, and WAI score. Individual and MSD‐related variables were no longer significant in the multivariable regression model (*p* > 0.05). Among organizational variables, working in ICUs, compared with EDs, was associated with lower levels of JS (*p* = 0.005). Likewise, employment in long‐shift schedules was additionally associated with reduced JS (*p* = 0.003). Finally, higher WAI scores were associated with increased JS (*p* < 0.001). The multiple regression for WAI included the following variables: sex, age, chronic conditions, cardiovascular disease, fitness for work with adjustments, physical activity, department, change of unit or role due to MSDs, body region affected by MSDs, and JS. Male sex was significantly associated with higher WAI score (*p* = 0.012). Conversely, a reduction in WAI was significantly associated with chronic condition (*p* = 0.001) and fitness for work with adjustment (*p* < 0.001). Organizational variables associated with a decrease in WAI included working in ICUs (*p* = 0.031) and experiencing a change of unit or role due to MSDs (*p* = 0.015). Among anatomical regions, hip (*p* = 0.009) and upper‐limb disorders (*p* = 0.006) were both associated with decreased WAI. Finally, an increase in JS was significantly associated with higher WAI scores (*p* < 0.001).

**TABLE 2:** Multiple regression between JS and WAI.

	JS	WAI
*Individual variables*		
Sex		
Female	/	Ref.
Male	/	1.28 (0.012)
Age		
< 40	Ref.	Ref.
40–55	−0.04 (0.647)	−0.67 (0.249)
> 55	0.143 (0.397)	−2.13 (0.007)
Chronic conditions		
No	/	Ref.
Yes	/	−3.14 (0.001)
Cardiovascular diseases		
No	/	Ref.
Yes	/	−0.68 (0.467)
Fitness for work with adjustments		
No	Ref.	Ref.
Yes	0.19 (0.136)	−2.58 (0.001)
Physical activity hours per week		
No	/	Ref.
< 1	/	0.15 (0.833)
1–3	/	0.25 (0.618)
> 3	/	0.72 (0.260)
*Organizational variables*		
Department		
ED	Ref.	Ref.
ICU	−0.38 (0.005)	−1.38 (0.031)
Long shift		
No	Ref.	/
Yes	−0.32 (0.003)	/
Change of unit/role due to MSDs		
No	Ref.	Ref.
Yes	−0.00 (0.989)	−1.60 (0.015)
Days of absence from work due to MSDs		
0	Ref.	/
1–7	0.13 (0.218)	/
8–30	−0.15 (0.354)	/
> 30	0.10 (0.635)	/
*Musculoskeletal disorders*		
Back		
No	Ref.	Ref.
Yes	−0.23 (0.051)	−0.65 (0.262)
Hips		
No	Ref.	Ref.
Yes	0.08 (0.524)	−1.69 (0.009)
Lower limbs		
No	Ref.	Ref.
Yes	0.05 (0.626)	−0.28 (0.586)
Upper limbs		
No	Ref.	Ref.
Yes	−0.08 (0.438)	−1.41 (0.006)
Questionnaire		
WAI	0.08 (< 0.001)	/
JS	/	2.22 (< 0.001)

*Note:* Values are reported as *β* coefficient (*p*‐value).

Abbreviations: JS, job satisfaction; WAI, Work Ability Index.

## 4. Discussion

This study aimed to identify individual, organizational, and MSD factors associated with JS and WA among critical care nurses and to explore the association between JS and WA. Findings suggest that JS is mainly associated with organizational factors, whereas WA showed associations with individual, organizational, and musculoskeletal factors.

Our sample reported overall moderate to above‐average JS, partly contrasting with previous evidence of lower JS in ICUs [8]. Therefore, it is plausible that ED nurses, although a minority of the sample, contributed to the higher mean JS. Our multivariate analysis showed JS was lower in ICU than ED nurses, aligning with previous studies [27]. Another significant organizational factor that emerged in our study and contributes to reducing JS is working long shifts. In line with previous studies, long shifts may contribute to lower JS [28], including in critical care settings [29]. Moreover, because long shifts are widely implemented in ICUs [30], their effects may further exacerbate the impact of the ICU work environment on JS.

Overall, the WAI score in this study was good with approximately half of the participants reporting excellent WA. Previous studies report moderate WA among ICU nurses [31] and good to excellent WA in ED nurses [32]. Consistently, our multivariable analysis showed that ICU work was associated with lower WA than ED work. This finding is consistent with the higher physical and psychological demands of ICU settings. Nevertheless, both settings present distinct occupational challenges that may influence overall WA [9].

Among organizational factors, changing roles or duties due to MSDs was associated with lower WA. Although such adjustments are recognized strategies to preserve WA among workers with MSDs [13], this association may indicate that nurses requiring these changes often experience more severe MSD‐related limitations or greater difficulty fulfilling previous job demands, rather than suggesting that role or duty changes themselves negatively affect WA [33]. This interpretation is consistent with previous evidence showing that fitness for work adjustments is often associated with lower WA, particularly among workers with health‐related limitations [34]. Among the main causes of fitness for work with adjustments are MSDs [9]. Hip disorders are frequently reported among critical care nurses and are associated with prolonged standing, heavy workload, and manual patient handling, reflecting the substantial physical demands of the role [9, 13, 31]. These disorders may contribute to reduced WA [13, 31]. Moreover, although the presence of upper limb disorders was consistently associated with lower WA in our study, the association between the DASH score and WA was not retained in the multivariate analysis. In the univariate analysis, the DASH score, which provides a more detailed assessment of upper limb‐related limitations in daily and work activities, was significantly associated with lower WA but was no longer statistically significant after adjustment in the multivariable model. This finding may indicate that the functional limitations captured by the DASH overlap with other health‐related or work‐related variables included in the model. Therefore, these results suggest that the presence of upper limb disorders may be independently associated with WA, whereas the specific level of disability measured by the DASH may not provide additional explanatory information when other factors are considered. Chronic conditions, which affected one‐third of our sample, may also necessitate fitness‐for‐work adjustments and are associated with reduced WA. Cardiovascular disease, when analyzed separately, did not show a significant association with WA in our study, in contrast to previous research among nurses and other healthcare professionals [35, 36]. Evidence specifically addressing the association between cardiovascular disease and WA among ICU nurses remains scarce, with available studies generally reporting a weaker association [31]. Thus, our findings suggest that reduced WA may reflect the cumulative or interacting effects of multiple chronic conditions rather than the effect of a single disease, with cardiovascular disease alone having little impact on work‐related or daily activities. Although chronic conditions [37] and MSDs increase with age [13], age was not significantly associated with WA in our study, contrary to previous findings [35]. Although age did not directly affect WA, an aging workforce with limited generational turnover [38] may experience declining functional abilities, which could affect WA. Moreover, male participants, though a small proportion of our sample, reported higher WA, consistent with previous studies [34], a finding reflecting occupational and societal factors [39].

Finally, WA and JS were significantly associated in both regression models, consistent with previous literature [10, 40]. Since, in our study, WA resulted from a wide range of factors, some nonmodifiable, such as chronic conditions, age, and MSDs, whereas JS is primarily shaped by potentially modifiable organizational factors, improving organizational conditions and enhancing JS may support and preserve nurses’ WA and related outcomes.

### 4.1. Linking Evidence to Action

The findings highlight the need for integrated strategies to sustain WA and JS of critical care nurses. A multidisciplinary, multilevel, and multicomponent approach, involving nurse leaders, managers, and occupational health professionals across primary, secondary, and tertiary prevention, is essential to promote well‐being, prevent MSDs, and support workforce sustainability [41]. Preventive strategies should prioritize unit‐level actions for nurses at risk of hip and upper‐limb disorders, including optimizing shift patterns, reducing prolonged standing and working hours, ensuring adequate recovery time, and strengthening training on the safe and consistent use of assistive devices. These measures should be complemented by proactive health surveillance for early MSD detection, alongside ergonomic improvements, task redesign, and targeted skills development. When symptoms arise, structured rehabilitation and work adjustments (e.g. modified duties) should be reinforced by follow‐up processes that help staff perceive these measures as supportive rather than restrictive. Overall, combining interventions such as task rotation and ergonomic workplace adaptations may help sustain WA and long‐term employability.

### 4.2. Strengths and Limitations of the Study

A key strength of this study is its novel insights into JS, WA, and MSDs among critical care nurses, supported by validated questionnaires that enhanced the reliability and comparability of the results. Nevertheless, some limitations should be acknowledged. First, the cross‐sectional design is an important limitation, as it cannot establish causal relationships. Therefore, the observed associations should be interpreted with caution, and longitudinal studies are needed to clarify the directionality of these relationships. Second, the use of self‐reported measures may have introduced reporting bias. In addition, as participants were asked to refer to their experiences over the previous 12 months, recall bias cannot be excluded. Data were collected between July and October 2022; therefore, the 12‐month reference period partly overlapped with the COVID‐19 pandemic, which may have affected JS and WA by increasing psychological distress, physical symptoms, and fatigue [42]. Third, because both predictors and outcomes were collected using the same questionnaire, potential common‐method bias should be considered. Finally, the sample was unbalanced, with a predominance of ICU nurses, limiting comparisons with ED nurses and potentially confounding organizational effects.

## 5. Conclusion

This study highlights that JS in critical care nurses is mainly influenced by organizational factors, while WA is shaped by a combination of individual, organizational, and musculoskeletal factors. The observed association between JS and WA underscores the importance of improving organizational conditions and implementing targeted occupational health strategies through a multidisciplinary, multilevel, and multicomponent approach.

Future studies could use longitudinal designs and directly compare critical settings to better understand how clinical environment, workload, and MSD factors influence JS and WA.

## Funding

This research received no specific grant from any funding agency in the public, commercial, or not‐for‐profit sectors. Open access publishing was facilitated by Universita degli Studi di Torino, as part of the Wiley ‐ CRUI‐CARE agreement.

## Conflicts of Interest

The authors declare no conflicts of interest.

## Supporting Information

Additional supporting information can be found online in the Supporting Information section.

## Supporting information


**Supporting Information** Supporting Information 1 presents the results of the univariate linear regression analyses examining the associations of individual, organizational, musculoskeletal, and questionnaire‐related variables with JS and WA.

## Data Availability

The data that support the findings of this study are available from the corresponding author upon reasonable request.
