# Supplementary material for: Organizational and Musculoskeletal Factors of Job Satisfaction and Work Ability Among Critical Care Nurses: A Multicenter Cross‐Sectional Study
Source: Nurs Res Pract. 2026 Sep 29;2026:4882421. doi: 10.1155/nrp/4882421 (PMC13624387; doi:10.1155/nrp/4882421)
Supplement: Supplementary file 1 [file NRP-2026-4882421-s001.docx]

**Supplementary 1:** *Univariate linear regressions between JS/WAI and included variables*

|  | **JS** | | **WAI** | |
| --- | --- | --- | --- | --- |
|  | **Coefficient** | **P. value** | **Coefficient** | **P. value** |
| **Individual variables** | | | | |
| **Sex** |  |  |  |  |
| Female | Ref. |  | Ref |  |
| Male | -0.002 | 0.983 | 1.406 | 0.051 |
| **Age** |  |  |  |  |
| <40 | Ref. |  | Ref. |  |
| 40-55 | -0.26 | 0.013 | -2.77 | 0.000 |
| >55 | -0.27 | 0.127 | -5.93 | 0.000 |
| **Cohabitation with a non-self-sufficient person** |  |  |  |  |
| No | Ref. |  | Ref. |  |
| Yes | 0.185 | 0.398 | -0.946 | 0.473 |
| **Chronic conditions** |  |  |  |  |
| No | Ref. |  | Ref. |  |
| Yes | -0.068 | 0.535 | -5.257 | 0.000 |
| **Cardiovascular diseases** |  |  |  |  |
| No | Ref. |  | Ref. |  |
| Yes | 0.052 | 0.800 | -4.501 | 0.000 |
| **Certified disability** |  |  |  |  |
| No | Ref. |  | Ref. |  |
| Yes | 0.021 | 0.938 | -2.341 | 0.143 |
| **Fitness for work with adjustments** |  |  |  |  |
| No | Ref. |  | Ref. |  |
| Yes | -0.032 | 0.013 | -6.093 | 0.000 |
| **Physical activity hours per week** |  |  |  |  |
| No | Ref. |  |  |  |
| < 1 | -0.173 | 0.330 | -0.015 | 0.988 |
| 1-3 | -0.129 | 0.280 | 0.937 | 0.190 |
| >3 | -0.039 | 0.797 | 1.729 | 0.051 |
| **Organizational variables** | | | | |
| **Department** |  |  |  |  |
| ED | Ref. |  | Ref. |  |
| ICU | -0.369 | 0.004 | -2.206 | 0.004 |
| **Long shifts** |  |  |  |  |
| No | Ref. |  | Ref |  |
| Yes | -0.178 | 0.087 | -0.112 | 0.857 |
| **Week working hours** | -0.009 | 0.521 | -0.143 | 0.116 |
| **Change of unit/role due to MSD** | -0.368 | 0.034 | -5.43 | 0.000 |
| **Absence from work due to MSDs** |  |  |  |  |
| No | Ref. |  | / |  |
| Yes | -0.31 | 0.003 | / |  |
| **Days of absence from work due to MSDs** |  |  |  |  |
| 0 | Ref. |  |  |  |
| 1-7 | -0.09 | 0.398 | / |  |
| 8-30 | -0.70 | **0.000** | / |  |
| > 30 | 0.54 | **0.012** | / |  |
| **Musculoskeletal disorders** | | | | |
| **Overall MSDs** | -0.193 | 0.083 | -2.99 | 0.001 |
| **Back** | -0.20 | 0.000 | -3.16 | 0.000 |
| **Hips** | -0.24 | 0.096 | -4.42 | 0.000 |
| **Lower limbs** | -0.19 | 0.083 | -2.86 | 0.000 |
| **Upper limbs** | -0.37 | 0.003 | -3.55 | 0.000 |
| **Questionnaire** | | | | |
| **DASH** |  |  |  |  |
| DASH-SF | -0.154 | 0.000 | -1.579 | 0.000 |
| DASH-W | -0.014 | 0.000 | -0.157 | 0.000 |
| DASH-SM | -0.002 | 0.638 | -0.044 | 0.185 |
| **WAI Overall Score** | 0.080 | 0.000 | / |  |
| < 28 | Ref. |  |  |  |
| 28-36 | 0.679 | 0.019 | / |  |
| 37-43 | 1.945 | 0.000 | / |  |
| > 43 | 1.777 | 0.000 | / |  |

JS= job satisfaction; WAI= work ability index
